# Seasonal variation in breast cancer diagnosis in Singapore

**DOI:** 10.1054/bjoc.2000.1654

**Published:** 2001-05

**Authors:** F Gao, D Machin, K-S Khoo, E-H Ng

**Affiliations:** Division of Clinical Trials and Epidemiological Sciences, National Cancer Centre, 11 Hospital Drive, Singapore 169610; Clinical Trials Research Unit, Institute of Primary Care & General Practice, University of Sheffield, Community Sciences Centre, Northern General Hospital, Sheffield S5 7AU, UK; Mount Elizabeth Medical Centre, Singapore 228510

## Abstract

This study investigates seasonality in the diagnosis of 3219 female breast carcinoma cases reported between 1995–8 in Singapore. There is little evidence of marked seasonal variation. Angular regression suggested that observed differences in peak diagnosis with respect to menopausal status, tumour size, ER and PR status may be chance. © 2001 Cancer Research Campaign http://www.bjcancer.com


British Journal of Cancer (2001) **84**(9), 1185–1187
© 2001 Cancer Research Campaign
doi: 10.1054/ bjoc.2000.1654, available online at http://www.idealibrary.com on IDEAL®

http://www.bjcancer.com

Short Communication

### Seasonal variation in breast cancer diagnosis in Singapore

**F Gao[1], D Machin[1,2], K-S Khoo[1] and E-H Ng[3]**

[1]Division of Clinical Trials and Epidemiological Sciences, National Cancer Centre, 11 Hospital Drive, Singapore 169610; [2]Clinical Trials Research Unit, Institute of Primary Care & General Practice, University of Sheffield, Community Sciences Centre, Northern General Hospital, Sheffield S5 7AU, UK; [3]Mount Elizabeth Medical Centre, Singapore 228510

**Summary** This study investigates seasonality in the diagnosis of 3219 female breast carcinoma cases reported between 1995–8 in Singapore. There is little evidence of marked seasonal variation. Angular regression suggested that observed differences in peak diagnosis with respect to menopausal status, tumour size, ER and PR status may be chance. © 2001 Cancer Research Campaign http://www.bjcancer.com

Seasonality in the presentation of breast cancer has been claimed in countries with distinct climatic seasons. Thus in northern latitudes, the peak of the seasonal variation in Israel occurred in Spring (Cohen et al, 1983), USA in May (Jacobsen and Janerich, 1977), while Southampton, England it was in June (Kirkham et al, 1985). In contrast, Galea and Blamey (1991) suggest in Nottingham, England there is 'no difference in frequency of tumour detection' – here May to August compared to September to April. On the other hand peaks for those tumours self-detected and requiring surgery occurred in Spring and late Autumn in the USA (Ross et al, 1997) with a similar pattern in Bulgaria (Dimitrov et al, 1998). In southern latitudes the peak of initial detection of breast cancer in Auckland, New Zealand occurred in December (Holdaway et al, 1990).

It has been suggested that the peak presentation may vary with patient characteristics. Thus Kirkham et al (1985) noted that the seasonality is more pronounced in premenopausal than post-menopausal women who peaked 3 months earlier in March. They also suggested that the small tumours (<3 cm diameter) were diagnosed 1 to 2 months earlier than the larger ones.

However, in none of these studies has the possibility been quantified that the seasonal pattern observed is an artifact of the confounding influence of referral patterns.

Singapore is a small island and this, as well as the health care system itself, facilitates open access to care. The 3.7 million population comprises peoples whose origins are mainly Chinese (78%), Malay (14%) or Indian (7%) (Lau, 1993). Being equatorial (latitude 1˚ north), the tropical climate has a relatively unchanging pattern over the year with daily temperatures ranging from 24 to 32˚C. As previous studies have all been conducted in countries with distinct weather patterns and with lower overall health care delivery system performance (WHO, 2000), evidence from Singapore may be particularly useful in examining the influence of any seasonal variation in breast cancer.

The Singapore Breast Cancer Registry identified all permanent residents of Singapore with malignant breast carcinoma from January 1995 to December 1998 and the corresponding medical and histology records were collected and reviewed. The date of diagnosis was taken as the day of surgery or when the malignancy was either clinically or histologically confirmed. These would usually be some days after the date of first presentation.

In addition, ethnicity (Chinese, Malay, Indian or others), date of birth, menstrual, oestrogen and progesterone receptor (ER, PR) status; tumour size and stage were recorded. Menopausal status was considered to be uncertain if patients had bilateral oophorectomy.

Data from each calendar year is standardized to 12 months of equal duration and the date of peak diagnosis is identified using the methods described in Machin and Chong (1998). The associated statistical significance was tested by Mardia $\chi^2$ statistic with 2 degrees of freedom (Mardia, 1972). The bootstrap technique (Fisher, 1993) was used to obtain a 95% confidence interval (CI) for the peak date using 1000 bootstrap samples of the same size as the number of patients under consideration.

The variation in date of peak diagnosis between different patient groups was explored using the methods of Fisher and Lee (1992). They describe how the regression of an angular variable, the day of diagnosis of breast cancer within the year, on a potential explanatory variable such as menopausal or receptor status can be made.

In total 3219 women were diagnosed with breast cancer over the 4 years. Figure 1 shows the frequency distribution of the date of diagnosis, presented as a rose diagram on a half monthly basis. There is no clear cut seasonal pattern although fewer cases are diagnosed in January and February (close to the Western and Chinese New Year festivals) and they appeared more numerous approximately 6 months later over the June to August period. The estimated peak (Table 1) of August 27 (95% CI July 14 to October 07) is statistically significant ($P = 0.015$) but of small magnitude ($R = 0.036$).

Table 1 shows the estimated peaks for groups based on patient and tumour characteristics recorded at presentation. It is clear, for example, that the peaks within the 4 ethnic groups are far from strong, and while those for the Chinese and Others coincide in August, that for the Malays is 3 weeks earlier in July whereas for the Indians, it is 2 months later in October. Only that for the Indian women is statistically significant ($P = 0.036$) but, with $R = 0.160$, this is not very marked.

*Received 27 July 2000*
*Revised 6 December 2000*
*Accepted 8 December 2000*

*Correspondence to:* F Gao

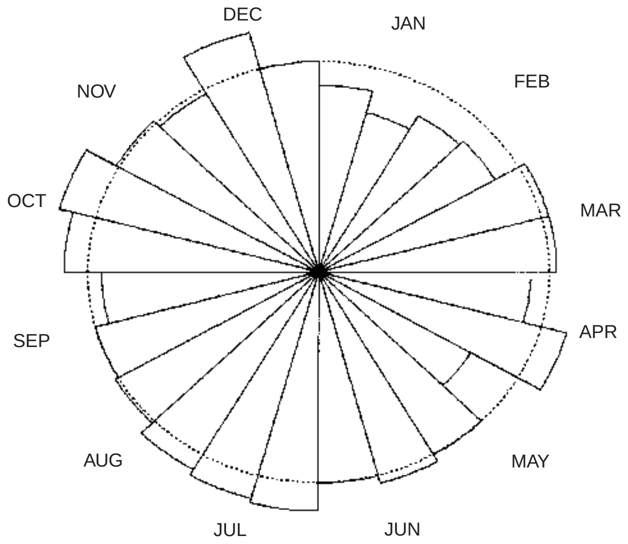

**Figure 1** Rose diagram of half-monthly diagnosis of female malignant breast cancer patients

There is only a 4-day difference in the peak dates for pre- and post-menopausal women. The peaks for ER- and PR-negative women essentially coincide in late August and early September respectively and both precede the peaks for the positive tumours by 1 month. These are all statistically significant but of relatively low magnitude. The close agreement in the results for ER and PR status arise since the status is the same for each in 78% of the women for which they are both observed.

The date of peak diagnosis for the women who have large sized tumours (≥1 cm) is late August in contrast to early December in those with the small tumours. However, the only statistically significant peak is for those with the larger tumours ($R$ = 0.049, $P$ = 0.002) but again this is of low magnitude.

Apart from Stage 0 patients who have an estimated peak in early July, the higher the stage the earlier the peak in diagnosis although only that for Stage IIA disease at September 25 is statistically significant ($R$ = 0.061, $P$ = 0.034).

Table 2 summarizes the associated regression analyses for the patient and tumour characteristics that are binary in nature. However, these analyses did not establish any statistically significant differences between the corresponding subgroups. For example, the observed 3-month difference in the peak dates of diagnosis for the different-sized tumours is not established as other than due to chance ($\beta$ = 1.112, 95% CI: – 0.81 to 3.04, $P$ = 0.26).

A clear drawback of this study (and most others in this area) is the uncertain relationship between date of diagnosis and date of onset of symptoms of breast cancer. The latter is more likely to be aetiologically important as the variable delays from first symptom to presentation and eventual diagnosis may depend on many factors. In our situation, the delay between onset of symptoms and presentation is uncertain but that between presentation and diagnosis is not likely to be great.

**Table 1** Summaries of seasonal variation by patient and tumour characteristics at presentation for all women

| | Number of cases | Corresponding calendar date | Magnitude of peak, $R$ | 95% CI | Mardia $\chi^2$ | $P$ |
|---|---|---|---|---|---|---|
| All | 3219 | Aug 27 | 0.036 | Jul 14–Oct 07 | 8.41 | 0.015 |
| Ethnicity | | | | | | |
| Chinese | 2743 | Aug 21 | 0.031 | Jun 21–Oct 10 | 5.31 | 0.070 |
| Malay | 307 | Jul 30 | 0.060 | Apr 04–Oct 21 | 2.22 | 0.33 |
| Indian | 130 | Oct 28 | 0.160 | Sep 09–Dec 13 | 6.65 | 0.036 |
| Others | 39 | Aug 20 | 0.130 | Mar 30–Dec 06 | 1.31 | 0.52 |
| Menopause | | | | | | |
| Pre | 1557 | Aug 19 | 0.026 | May 22–Jan 24 | 2.10 | 0.35 |
| Uncertain | 264 | | | | | |
| Post | 1398 | Aug 23 | 0.036 | Jun 13–Nov 04 | 3.59 | 0.17 |
| ER | | | | | | |
| Negative | 1061 | Sep 01 | 0.084 | Jul 27–Sep 30 | 14.86 | 0.0006 |
| Not done | 732 | | | | | |
| Positive | 1426 | Oct 02 | 0.056 | Aug 26–Nov 15 | 8.98 | 0.011 |
| PR | | | | | | |
| Negative | 1308 | Aug 31 | 0.071 | Jul 27–Oct 02 | 13.07 | 0.002 |
| Not done | 779 | | | | | |
| Positive | 1132 | Oct 04 | 0.076 | Sep 09–Nov 19 | 12.96 | 0.002 |
| Tumour (cm) | | | | | | |
| <1 | 338 | Dec 06 | 0.030 | Jul 16–May 06 | 0.60 | 0.74 |
| ≥ 1 | 2572 | Aug 30 | 0.049 | Jul 27–Oct 04 | 12.20 | 0.002 |
| Not done | 309 | | | | | |
| Stage | | | | | | |
| 0 | 276 | Jul 05 | 0.036 | Feb 10–Dec 08 | 0.70 | 0.71 |
| I | 770 | Sep 12 | 0.042 | Jun 13–Dec 11 | 2.68 | 0.26 |
| IIA | 922 | Sep 25 | 0.061 | Aug 09–Nov 15 | 6.76 | 0.034 |
| IIB | 662 | Aug 04 | 0.034 | Apr 01–Nov 23 | 1.52 | 0.47 |
| III | 208 | Jun 18 | 0.039 | Jan 30–Dec 04 | 0.63 | 0.73 |
| IV | 199 | Apr 16 | 0.026 | Nov 07–Oct 07 | 0.26 | 0.88 |
| Not done | 182 | | | | | |

 *© 2001 Cancer Research Campaign*

**Table 2** Regression coefficients for differences in peak date of diagnosis for selected patient and tumour characteristics at presentation for all women

| Variable | | Date of peak | Number of cases | Regression coefficient, β | Standard error | *P* |
|---|---|---|---|---|---|---|
| Menopause | Pre | Aug 19 | 1557 | 0 | | |
| | Post | Aug 23 | 1398 | 0.030 | 0.425 | 0.94 |
| ER | Negative | Sep 01 | 1061 | 0 | | |
| | Positive | Oct 02 | 1426 | 0.275 | 0.227 | 0.23 |
| PR | Negative | Aug 31 | 1308 | 0 | | |
| | Positive | Oct 04 | 1132 | 0.302 | 0.214 | 0.16 |
| Tumour size (cm) | ≥ 1 | Aug 30 | 2572 | 0 | | |
| | <1 | Dec 06 | 338 | 1.112 | 0.983 | 0.26 |

The overall health care system in Singapore is ranked very highly (WHO, 2000) and provides relatively open access to care although individuals are less likely to self refer during the span of the New Year (December to February) festivities. In addition, the tropical climate is of an essentially unchanging pattern over the year. For both these reasons a major seasonal component in the diagnosis of breast cancer (whether induced by climatic changes or referral) would not be anticipated. Thus the fewer cases in January and February and the consequential peak of small magnitude in August reflect self-referral patterns alone and not the presence of an aetiological determinant. Likewise, the corresponding seasonality reported in other studies may be enhanced (or obscured) by local referral characteristics, perhaps leading to a false indication of an underlying climatic component which might, for example, influence hormone activity as has been conjectured (Cohen et al, 1983; Mason et al, 1985).

When seasonality between patients with different characteristics is compared, our findings are not always consistent with previous studies. Thus while Kirkham et al (1985) reported peak presentation for pre-menopausal women was 3 months earlier than for post-menopausal, they differed by only 4 days in Singapore (Table 1).

The regression methodology introduced, analogous to that used routinely in other areas of clinical research, potentially allows a more detailed investigation of possible seasonal patterns. In this study, these methods suggested observed differences between groups might be no more than chance. This will not necessarily be the case in other geographical locations.

If a climatic component played a major role in the development of breast cancer, then one might anticipate some gradient between studies ranging from northern (and southern) latitudes to the equator and perhaps similarities between those of common latitude but differing longitude. One would anticipate little effect at the equator as we have observed. The findings of certain other studies may have been distorted by different health care delivery systems and other confounding variables. Studies reported to date show no such gradient but the lack of standardization in the identification of case presentation dates and reporting details make the true position unclear. To overcome these shortcomings, a coordinated and prospective study using individual dates of onset, patient specific and health care delivery details encompassing subjects from many latitudes and longitudes is required to reveal the extent of a climatic component, if any, in the aetiology of breast cancer.

### ACKNOWLEDGEMENTS

We would like to thank the National Medical Research Council, Singapore for funding the Singapore Breast Cancer registry and Ho Soo Leng for carefully collecting and collating the data.

### REFERENCES

Cohen P, Wax Y and Modan B (1983) Seasonality in the occurrence of breast cancer. *Cancer Res* **43**: 892–896

Dimitrov BD, Shangova-Grigoriadi S and Grigoriadis ED (1998) Cyclicity in variations of incidence rates for breast cancer in different countries. *Folia Med (Plovdiv)* **40**: 66–71

Fisher NI (1993) *Statistical Analysis of Circular Data*. Cambridge University Press: Cambridge

Fisher NI and Lee AJ (1992) Regression models for an angular response. *Biometrics* **48**: 665–677

Galea MH and Blamey RW (1991) Season of initial detection of breast cancer. *Br J Cancer* **63**: 157

Holdaway IM, Mason BH, Marshall RJ, Neave LM and Kay RG (1990) Seasonal change in the concentration of progesterone receptor in breast cancer. *Cancer Res* **50**: 5883–5886

Jacobsen HK and Janerich DT (1977) Seasonal variation in the diagnosis of breast cancer. (Abstract) *Proc Am Assoc Cancer Res* **18**: 93

Kirkham N, Machin D, Cotton DWK and Pike JM (1985) Seasonality and breast cancer. *Eur J Surg Oncol* **11**: 143–146

Lau KE (1993) *Census of Population 1990*. Department of Statistics: Singapore

Machin D and Chong SF (1998) On the detection of the seasonal onset of disease. *J Epid Biostats* **3**: 385–394

Mason BH, Holdaway IM, Mullins PR, Kay RG and Skinner SJ (1985) Seasonal variation in breast cancer detection: correlation with tumour progesterone receptor status. *Breast Cancer Res Treat* **5**: 171–176

Mardia KV (1972) *Statistics of Directional Data*. New York: Academic Press

Ross JA, Severson RK, Davis S, Stanford JL and Potter JD (1997) Seasonal trends in the self-detection of breast cancer: indications from the Cancer and Steroid Hormone (CASH) study. *Breast Cancer Research and Treatment* **42**: 187–192

WHO (2000) *World Health Report 2000: Health Systems: Improving Performance*. World Health Organization, Geneva, 151–155

*© 2001 Cancer Research Campaign* 

